# Analysis of Medico-Legal Complaint Data: A Retrospective Study of Three Large Italian University Hospitals

**DOI:** 10.3390/healthcare11101406

**Published:** 2023-05-12

**Authors:** Nicola Di Fazio, Matteo Scopetti, Giuseppe Delogu, Raffaele La Russa, Federica Foti, Vincenzo M. Grassi, Giuseppe Vetrugno, Francesco De Micco, Anna De Benedictis, Vittoradolfo Tambone, Raffaella Rinaldi, Paola Frati, Vittorio Fineschi

**Affiliations:** 1 Department of Anatomical, Histological, Forensic and Orthopaedic Sciences, Sapienza University of Rome, 00128 Rome, Italy; 2 Department of Clinical and Experimental Medicine, University of Foggia, 71122 Foggia, Italy; 3 Risk Management Unit, Fondazione Policlinico Universitario “A Gemelli” IRCCS—Legal Medicine, Department of Health Surveillance and Bioethics, Università Cattolica del Sacro Cuore, 00168 Rome, Italy; 4 Research Unit of Bioethics and Humanities, Department of Medicine and Surgery, Università Campus 12 Bio-Medico di Roma, Via Alvaro del Portillo, 21, 00128 Roma, Italy; 5 Fondazione Policlinico Universitario Campus Bio-Medico, Via Alvaro del Portillo, 200, 00128 Roma, Italy; 6 Research Unit of Nursing Science, Department of Medicine and Surgery, Università Campus Bio-Medico di Roma, Via Alvaro del Portillo, 21, 00128 Roma, Italy

**Keywords:** clinical risk management, medical liability, patient safety indicator, performance indicator, cost-effectiveness, adverse event, national rules and laws in clinical risk management

## Abstract

(1) Background: Identifying hospital-related critical, and excellent, areas represents the main goal of this paper, in both a national and local setting. Information was collected and organized for an internal company’s reports, regarding civil litigation that has been affecting the hospital, to relate the obtained results with the phenomenon of medical malpractice on a national scale. This is for the development of targeted improvement strategies, and for investing available resources in a proficient way. (2) Methods: In the present study, data from claims management in Umberto I General Hospital, Agostino Gemelli University Hospital Foundation and Campus Bio-Medico University Hospital Foundation, from 2013 to 2020 were collected. A total of 2098 files were examined, and a set of 13 outcome indicators in the assessment of “quality of care” was proposed. (3) Results: From the total number, only 779 records (37.1%) were attributable to the categories indexable for the present analysis. This data highlights how, following a correct and rigorous categorization of hospital events, it is possible to analyze these medico-legal aspects using a small number of indicators. Furthermore, it is important to consider how a consistent percentage of remaining events was difficult to index, and was also of poor scientific interest. (4) Conclusions: The proposed indicators do not require standards to be compared to, but provide a useful instrument for comparative purposes. In fact, in addition to comparative assessment between different business realities distributed throughout the territory, the use of outcome indicators allows for a longitudinal analysis evaluating the performance of an individual structure over time.

## 1. Introduction

Risk management represents the main instrument through which Healthcare Institutions can supervise healthcare services, as well as improve their efficiency [1,2], in order to develop targeted improvement strategies and invest available resources in a proficient way [3,4,5].

Historically, the concept of implementing quality has often been hampered by the lack of objective, measurable, and comparable data: in this context, information tools, generically called indicators, can help administrators and health professionals in highlighting the criticalities of the system to direct priority choices [6].

Over the last few years, initiatives aimed at advertising rankings of hospitals or individual professionals have spread [7,8], defined according to the clinical results obtained, and documented through indicators [9,10,11]. In the current Italian health system [12,13], accreditation of health facilities is an indispensable prerequisite for any health facility to become an effective provider of remunerated services [14]. Accreditation, as configured by national legislation, represents a qualified tool for selecting providers on behalf of the National Health Service, characterized by the necessary correspondence to a series of requirements [15]. These are directly related to expected quality levels, as well as to the temporariness of their adequacy recognition. This system aims to promote the process of continuous improvement of performance quality, organization efficiency, and use of resources and training [16,17].

As shown in the literature [18,19,20,21,22,23], individual hospitals that adopted such initiatives have developed greater attention to the quality of care, or rather, to those aspects of care that were specifically the subject of public evaluation. “Quality of care” refers to a collection of different dimensions related to various aspects of the work of both healthcare professionals and organizations [22,24,25,26,27,28,29], defined by a criteria of accessibility, continuity, effectiveness, efficiency, clinical and organizational appropriateness, safety [27], timeliness, centrality of patients [28], and human resources development [22,29].

Scientific evidence identifies indicators as the best tool to measure performance values [30,31]. Defined as “a synthetic measure, generally expressed in quantitative form, coinciding with a variable or composed of several variables, capable of assuming the trend of the phenomenon to which it refers” [32], these variables are characterized by high information content, capability to allow a rapid evaluation of phenomena that are sometimes very complex, and to provide pivotal elements in operational decisions; furthermore, they can be used to compare phenomena over time (at different moments) and in space (in different realities), or concerning a goal that is to be achieved or maintained.

It can, therefore, be quantitative, ordinal, or semi-quantitative or -qualitative. To establish a set of indicators, the structure in which they are inserted, expressing the value of a specific characteristic, should be clarified. Homogeneous groups of indicators are included within the same index, which is a more complex tool in which multiple components (indicators) can interact with each other in different ways, through one or more mathematical operators. The index provides an easier parameter, which is more immediate to understand. Similarly, indices can be included in a macro-group called “dimension”, referring to that thematic area that aggregates performance indicators about the homogeneity of the phenomena they intend to measure and evaluate [33]. Within dimensions, sub-dimensions are provided to simplify the analysis of investigated phenomena.

To achieve effective and efficient use of indicators, however, it is necessary to constantly remember their limits. In fact, it is not possible to affirm anomalies or important variations of a phenomenon based only on the indicator’s alteration compared to its reference value (standard). At least a certain number of elements must be anomalous to correctly identify, with an acceptable degree of certainty, a real alteration in processes or phenomena. Therefore, the construction of good-quality indicators is fundamental for both their validity and use. To guarantee indicators’ completeness, validity, and precision, controls should be programmed, monitoring both archived data and newly collected ones [34].

For an indicator to provide quality of care information, it must be compared, either with values considered as reference (standard), or with values of the same unit under examination obtained at different times, or with those of other observation units [35]. Standards, depending on the role they play, can be summarized as follows: starting standards (at the beginning of the monitoring activity), improvement standards (objective to be achieved), quality standards (the best possible quality), and accreditation standards (mandatory levels for the acquisition of specific authorizations).

Three main uses of indicators can be distinguished [36,37]:

- Scientific and research purposes;
- Internal evaluation and improvement within an organization (continuous quality improvement and internal evaluation of healthcare organizations);
- Evaluation and improvement of an organization in relation to the outside world (national indicators). This makes it possible to compare similar organizations (benchmarking), and is also widely applied during accountability and accreditation processes [38,39,40].

In this evolutionary context, healthcare-associated performance indicators constitute a powerful tool, through which decision-makers can grasp initial conditions of a system, identify quality and safety issues, and quantify reasonably achievable objectives [41,42,43,44]. Moreover, indicators allow to verify the correspondence between results obtained and those expected, and to measure the impact of carried-out activities [45]. A further objective of performance indicators analysis would be to hinder the phenomenon of defensive medicine that affects many specialist fields of medicine [46]. It is important to underline that monitoring actions are not always easy from a technical-methodological point of view [47,48]. Healthcare is a multidimensional product, and there are no stable relationships, either between overall healthcare expenditure and resources supply, nor between resources and healthcare services (due to management efficiency diversity), nor, finally, between services and health outcomes [49].

Therefore, it is necessary to focus on one or more sets of indicators capable of providing information regarding a plurality of phenomena ranging from allocation of physical resources to expenditure [50]. The construction of this integrated and balanced system of indicators presents further difficulties. The plurality of recipients determines multiple interests, which are not necessarily convergent. Type and quality of produced information, ways of presenting data, level of complexity, and disaggregation adopted in the process of producing indicators may, therefore, differ, depending on the user to whom the selected system of indicators is addressed.

Finally, emphasis on healthcare assessment systems is progressively shifting from simple spatial-temporal comparisons to more complex aspects related to benchmarking, extending to the clinical-epidemiological field [51].

Traditionally, the analysis of health services and their quality is based on Donabedian’s conceptual model, which provides three indicator topologies [52,53,54,55,56]:

- Structure indicators;
- Process indicators;
- Outcome indicators.

Process measures are essential in providing guidance to professionals on how to modify a path to improve its impact on patient care outcomes [57,58].

Notably, outcome indicators document a change in clinical (health, mortality, morbidity), economic (direct and indirect costs), and humanistic (quality of life, user satisfaction) care standards, and, furthermore, they tend to directly highlight results and are easier to understand [59,60].

Nonetheless, a widely stressed concept is that an adequate use of indicators lies in their actionability, which can be summarized in the need to be simultaneously “fit for purpose” and “fit for use” [61].

Therefore, the first goal of our analysis was to develop a system for assessing and monitoring the quality of services provided by 3 large Italian health facilities. The application of these concepts to the clinical and managerial reality of large healthcare facilities is a novelty in the scientific panorama, as it has not been possible to find articles similar to the present in the literature.

Therefore, the selection of categories of indicators that maximize the performance improvement process was based on the analysis of the 6 dimensions of quality: safe, effective, patient-centered, timely, efficient, and equitable care [62]. On the basis of these requirements, the proposed data represent an absolute novelty in the clinical and managerial landscape of healthcare facilities; moreover, in addition to the “photographic” value in their ability to objectively show a present scenario, they possess the potential for a future prospective analysis, and for a possible comparison between international structures, in order to assist a wide-range improvement process on issues of absolute relevance (e.g., fight against antimicrobial resistance).

## 2. Materials and Methods

A set of performance indicators was developed based on data from the medical-legal claims of Umberto I General Hospital, Agostino Gemelli University Hospital Foundation and Campus Bio-Medico University Hospital Foundation, from 1 January 2013 to 31 December 2020. Starting from July 2018, a unification and normalization operation of existing reports, relating to the management of civil litigation operated by the Legal Medicine Units of Umberto I General Hospital, Agostino Gemelli University Hospital Foundation and the Department of Clinical Affairs of Campus Bio-Medico University Hospital Foundation, was carried out.

Umberto I General Hospital is a mixed welfare and educational institution, equipped with a second-level Emergency Department and 1235 ordinary beds. In the last year of operation, it carried out about 41,000 hospitalizations, while more than 140,000 patients accessed the emergency room. It also provides 1,000,000 services per year between instrumental diagnostics and medical examinations, 900,000 laboratory tests, 20,000 histopathological investigations, and as many radiological investigations.

Agostino Gemelli University Hospital Foundation is both a welfare and an educational institution. Eight clinical and research departments involve 113 medical care units, including 86 complex operating units, 27 simple operating areas, and 1536 beds. In one year, 215 organ transplantations were performed, 94,509 patients were discharged, and 83,419 patients accessed the emergency room.

Campus Bio-Medico University Hospital Foundation is a mixed welfare and educational institution, equipped with an Emergency Department, 296 ordinary beds, and more than 60 medical care units. In the last year of operation, it carried out about 28,700 hospitalizations and 530,000 outpatient treatments, while more than 11,600 patients accessed the emergency room (Table 1).

The collected material was initially heterogeneous in content and structure, and provided for direct consultation of 2098 files. All cases included in the database presented the following features:

- they were all categorized according to the International Classification for Patient Safety (ICPS) system [63], with particular attention to the following parameters: age, gender, unit involved, event date and type, outcome (for patient and structure involved);
- economic quantification of the request, technical opinion, risk of loss, eventual amount paid;
- chronological specification of the judicial (or extrajudicial) phase.

Later, the most significant data from the collected cluster of clinical records were extrapolated. In our study, the high number of cases ensures a high level of representativeness compared to the reference population. All data and information acquired were collected and organized digitally on a Microsoft Excel single sheet, through an entry string structured according to descriptive indicators of event and damage, or presumed damage, of subject, as well as parameters of medico-legal evaluation.

Following the first phase of database homogenization obtained through individual review of cases in close collaboration with legal offices, Excel calculation functions were applied through the construction of “pivot” tables.

Finally, results were summarized in graphical form and using summary tables. Collected data were distributed according to a wide qualitative and quantitative variability, especially about opening date of the claim and corresponding event, which is why our reference sample may be slightly inconstant depending on the case, which will be specified from time to time.

Although this study was conducted as multicentric, the ultimate aim is not the comparison between healthcare-related litigation data from the structures involved. Therefore, the data presented will be exposed by combining the individual values found, thus considering the structures in question as a single group.

### 2.1. Indicators Set

The set of indicators was chosen to evaluate health facilities’ performance. The choice was made possible by the application of the Process Analysis Method (PAM) [64], which demonstrated its suitability for performance evaluation of Umberto I General Hospital in a previous experience by Scopetti et al. [65]. The implementation of the PAM system consists of five phases:

- Phase I—Overview of the claims. During this phase, all files were examined;
- Phase II—Definition of performance in claims management, established as the contribution in terms of results of a subject to the achievement of an objective;
- Phase III—Setting system limits. The spatial criterion consisted of all claims based on civil and health liability, while the temporal one was the limitation of claims to the period 2013–2020;
- Phase IV—Setting up the performance scoreboard. In this phase, a set of indicators was selected and standardized for name, definition, and calculation formula. Notably, all indicators were validated against internationally recognized benchmarks, such as the Agency for Healthcare Research and Quality (AHRQ);
- Phase V—Verification by reviewing all data, measures, and indicators.

Moreover, since the aim of this study was to ensure an improvement in quality of care, the choice of indicators was guided by their scientific validity and, as well as their measurability.

In particular, the specific dimension identified was “clinical setting”. In this dimension, the index used was “quality of care”. All indicators were chosen on the basis of the most representative claim-related events that occurred within the selected structures, and were expressed in the form of rates (ratio between two numerical quantities) (Table 2).

In contrast to what was done previously [65], no internal standard was applied to the analysis of the identified indicators. Internal standard refers to the ratio between average of claims reported per year and the cumulative number of annual hospitalizations [64]. Such an instrument is particularly useful to monitor the trend of standardized variables over time, but it reaches maximum significance in the event that the data come from a homogeneous source, and not from multiple structures. In fact, their variability would prevent a value from being correctly evaluated based on a pre-set threshold dependent on the values obtained. Since the objective of this paper is to observe the trend of claims on a larger scale than the individual structure, the use of KPIs alone can, therefore, be considered sufficient.

### 2.2. Bias Risk Assessment

Since the present analysis constitutes the first attempt to start a line of applied research on the use of performance indicators in a healthcare setting, an approach based on eliminating the major complexities related to data extraction has been adopted, as well as on the use of a numerical and graphic language based on simplicity and immediate understanding of the results shown. The use of data relating to the outcome of health services interfaces perfectly with this purpose, but exposes the analysis conducted to the risk of not considering equally important elements, including the compliance of companies with structural and accreditation requirements, or of not deepening the adequacy of care and treatment pathways. These aspects, extremely heterogeneous from each other, require a dedicated disquisition.

Furthermore, the events considered in this analysis consist of the claims reported in the period of interest, and this entails a limited view of the events resulting from legal disputes; the number of claims does not necessarily correspond to the total number of adverse events, which, however, can be ignored for numerous reasons, including failure to report to the health management in the form of an audit.

The multicentric approach adopted in this paper allows, in any case, to limit several critical elements determined by the influence that the structural and welfare elements of each structure have in terms of outcome; moreover, from a management point of view, based on the analysis of clinical efficiency and cost effectiveness, the monitoring of the trend of claims certainly constitutes an optimal approach to understanding the degree of quality perceived by patients and the related economic aspect.

## 3. Results

After collecting, analyzing, and subsequently indexing all files, a preliminary consideration is that a slight fluctuation in the incidence of the single categories of events identified was observed among the structures investigated, but it should be emphasized that, as previously stated, the comparison between the single centers was not an objective of this paper, which, on the contrary, was based on the analysis of a database as broad and representative as possible of the Italian national scenario. However, the work of merging the data, and careful observation of the same, revealed characteristics of homogeneity, so, the extrapolated considerations can be considered free from “disequilibrium” biases.

**Figure 1:**
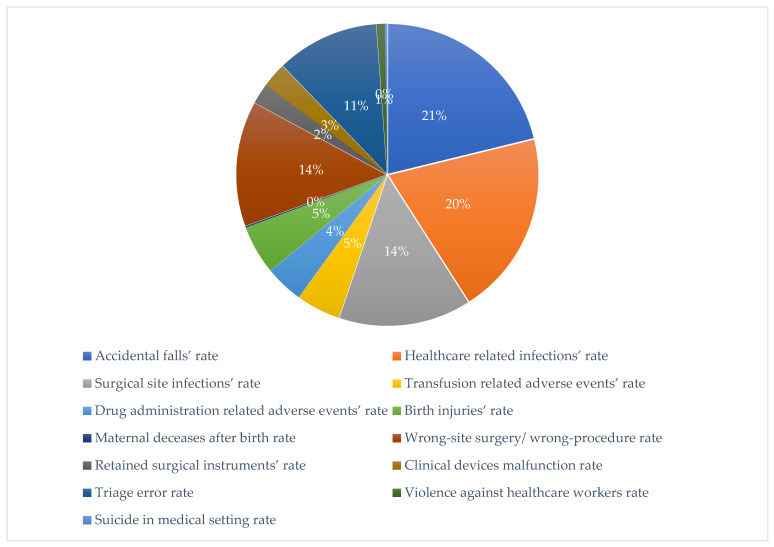
“Quality of care” analysis of Umberto I General Hospital, Campus Bio-Medico University Hospital Foundation and Agostino Gemelli IRCCS University Hospital Foundation, from 2007 to 2020.

From the total number, only 779 records (37.1%) were attributable to the categories indexable for the present analysis. Obtained results are represented and summarized in the following graphic representation (Figure 1).

It can be preliminarily stated that, as shown in the graphic above, the type of event most represented within the structures considered is healthcare-related accidental fall (21%), with a slightly higher incidence than healthcare-associated infections (HAI, 20%). Below, the two most represented categories after the previous ones are that of surgical site infections (SSI) and wrong-site surgery or wrong procedure (14% of incidence for both). These data partially follow what was observed in a previous experience of litigation data, relating only to the Umberto I General Hospital, underlining the high incidence of events related to HAIs and the impact of the surgical sphere on the total number of claims [66]. The events characterized by the lowest percentage of incidence were the acts of violence against healthcare workers (1%), suicides in medical setting, and cases of maternal decease after birth (both with an incidence of less than 1%).

**Figure 2:**
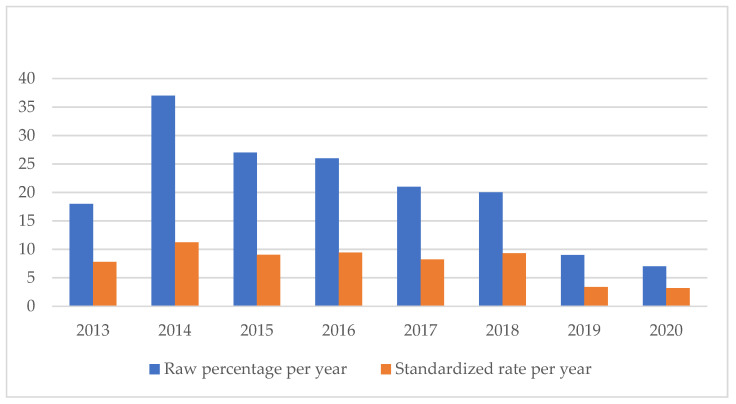
Accidental falls rate.

**Figure 3:**
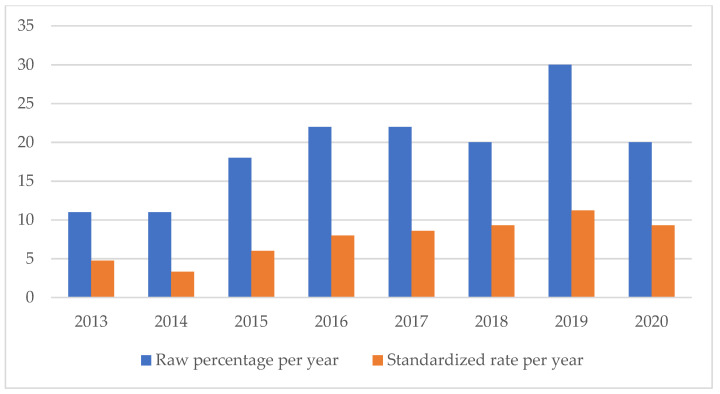
Healthcare-related infections rate.

**Figure 4:**
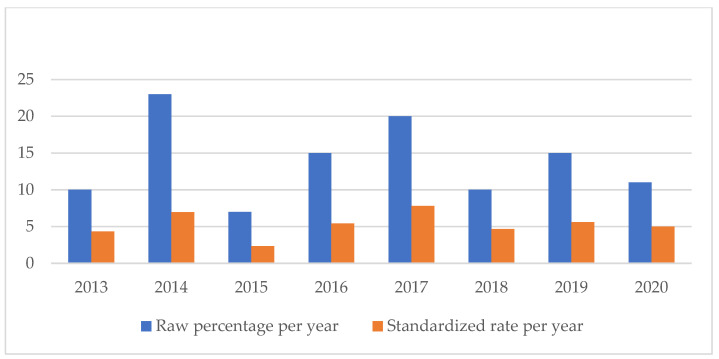
Surgical site infections rate.

**Figure 5:**
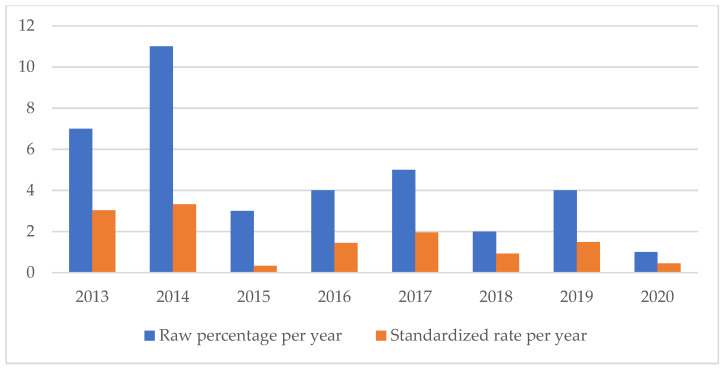
Transfusion-related adverse events rate.

**Figure 6:**
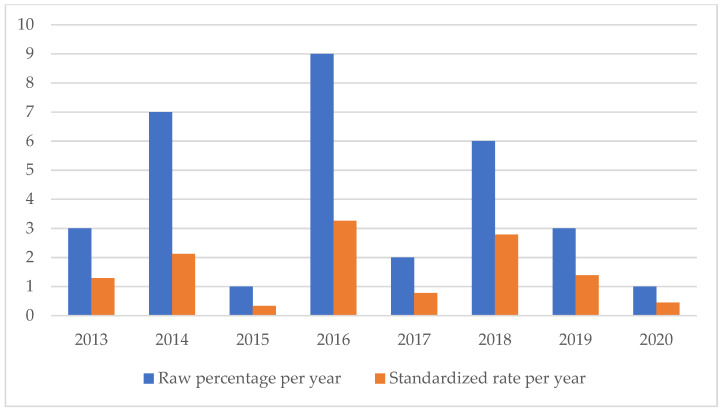
Drug administration-related adverse events rate.

**Figure 7:**
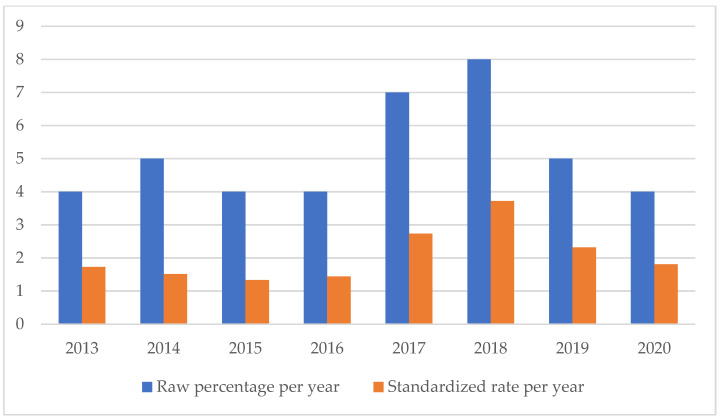
Birth injuries rate.

**Figure 8:**
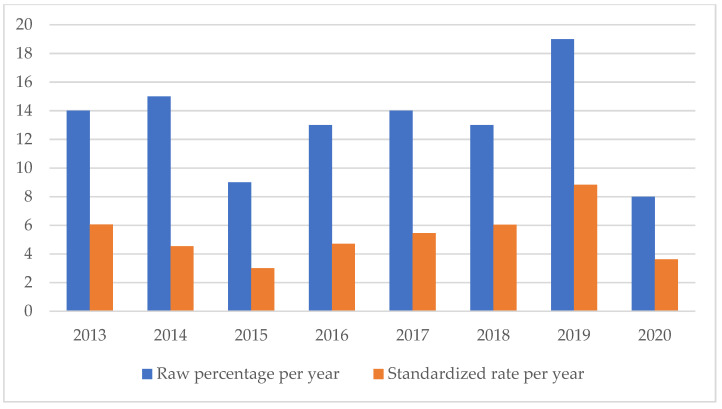
Wrong-site surgery/wrong procedure rate.

**Figure 9:**
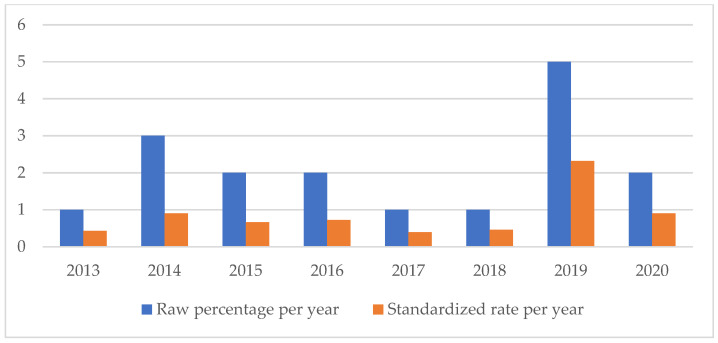
Retained surgical instruments rate.

**Figure 10:**
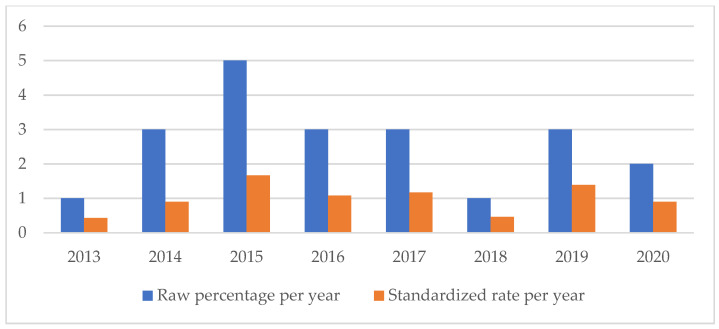
Clinical devices malfunction rate.

**Figure 11:**
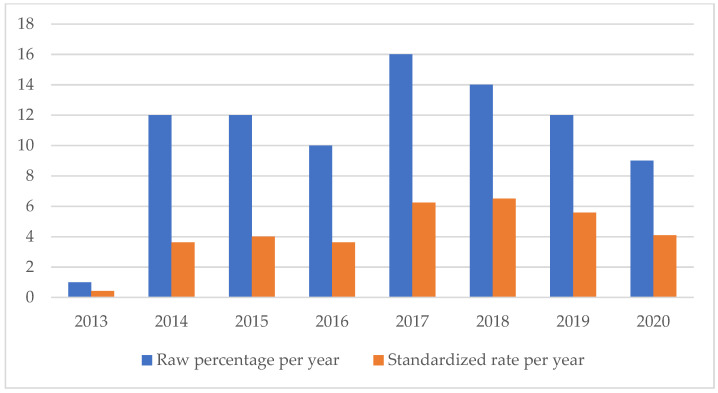
Triage error rate.

An analysis of the temporal trend of the events analyzed made it essential to standardize the total number of claims per year (Figure 2, Figure 3, Figure 4, Figure 5, Figure 6, Figure 7, Figure 8, Figure 9, Figure 10 and Figure 11). This made it possible to observe a consistent deflationary trend of claims related to accidental falls in healthcare settings and transfusion-related adverse effects, which reflects adequate organizational and procedural management of these events by examined healthcare facilities. This result takes on fundamental importance when compared with the absolute number of events, which, instead, shows an increasing trend over time: the importance of an adequate KPI, in this case, lies precisely in its ability to highlight how the number of specific adverse events compared to the total show a decreasing trend as an index of increase in the quality of care, with respect to the chosen item.

On the other hand, the SSIs are maintaining a stable trend during the selected period (Figure 4), highlighting the possibility of further preventive interventions. In addition, the number of HAIs over time appears to have increased, highlighting the need for more stringent measures against an entity that is assuming the character of a global emergency. Even an event of a psychiatric nature, such as suicide, has been the subject of our study through the elaboration of an appropriate indicator.

**Figure 12:**
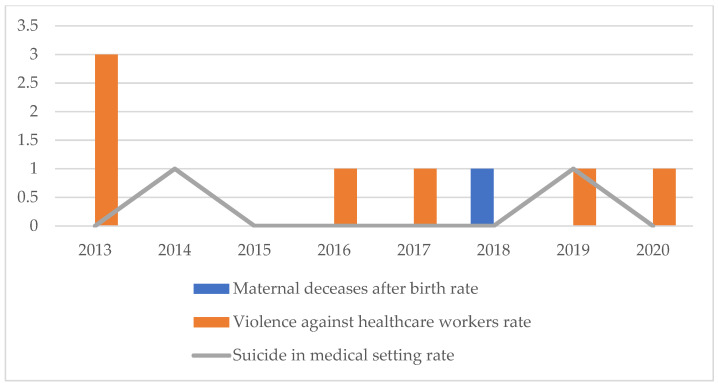
Maternal decease after birth rate, violence against healthcare workers, suicide in medical setting rate.

Finally, it is necessary to specify that no further investigations of a technical-statistical nature have been carried out about the three less-represented categories, since the numbers have not reached sufficiency to allow any statistically significant estimates (Figure 12).

However, it is significant to note how acts of violence against healthcare personnel saw a peak incidence in 2013 (3 episodes), followed by two years without such events, and, subsequently, by single episodes recorded per year; despite the smallness of the numbers presented, the reversal of the initial trend can still be interpreted positively in terms of adaptation of the structures to the needs of the patients, in terms of organization, communication, and quality of the service offered. On the other hand, the trend of suicides and the single episode of maternal death after birth can also be considered as anecdotal episodes and are, as such, worthy of further study, but without the need to proceed with further analytical disquisitions.

## 4. Discussion

Our study moves from a methodological approach, strongly oriented to scientific and doctrinal principles of academic forensic medicine within hospital organizational and professional systems, to manage health liability litigation [66]. The ethical dimension of clinical risk management was also considered in the choice of a multicentric setting that starts from shared experience to seek scientific truth, according to the ethics of a “Job Well Done” [67].

Moreover, an accurate analysis of litigation may help hospital systems to make process indicators, for example, extending the application of predictive tools widely used in the insurance world to the evaluation of incident reporting [68].

The objective behind the study was twofold. First, information was collected and organized for internal company reporting, regarding civil litigation that has been affecting the hospital, to relate results obtained with the phenomenon of medical malpractice on a national scale. Secondly, performance indicators were produced, allowing to carry out a standardized assessment of the clinical dimension of the structure. Even the production of an indicator for the suicide event is indicative of how important it is to assess the quality of a healthcare structure that requires personalized medical interventions for the patient [69,70,71].

Therefore, the present analysis highlights how, following a correct and rigorous categorization of hospital events, it is possible to analyze these medico-legal aspects using a small number of indicators.

On the other hand, it is important to consider how a non-negligible percentage of events on the total (1319 out of 2098 selected files) was difficult to index, being constituted of events such as theft or damage to property. These events certainly play a marginal role within the present discussion, since they are phenomena not directly linked to the treatment process, and are, therefore, of poor scientific interest in the present context. Furthermore, since they mainly depended on the structural and managerial set-up of the individual structures, these aspects are highly uneven among the analyzed centers. Despite this, they can still be categorized and analyzed for purposes other than that of the present investigation, so these aspects will be examined separately.

Among specific scientific literature, many studies apply process indicators in the context of performance assessments. The organizational and decision-making process that follows compares results with desirable values for each indicator, thus leading to corrective actions. As a result, those parameters that were found to be non-compliant are modified and implemented. In the meantime, company performance improvement programs are defined and priorities and margins for improvement identified.

However, these indicators, while potentially able to predict an improvement in care outcomes, do not provide information on the outcomes of care (outcomes). For this reason, we propose a set of 13 outcome indicators in the assessment of “quality of care”, which do not need a comparative standard for a longitudinal analysis.

Since there are no previous studies evaluating outcome indicators in a clinical setting, we suggest a set of 13 indicators. To address the clinical performance of the analyzed healthcare facility, our study focused on “quality of care”, and, therefore, on those outcome indicators related to this specific index. The elaboration process for all the indicators we chose moves from a thorough analysis of the most recurrent claims in the healthcare setting to their economic impact on the healthcare structure, and, further, to the evidence of possible corrective actions to reduce their frequency.

Several results that have emerged are highly encouraging. The first is certainly constituted by the homogeneity found in the classes of indicators identified among the structures being analyzed, which allows us to hypothesize the possibility of using these indicators on a large scale (e.g., city, province, region, nation, continent) in order to build a surveillance network of adverse clinical events that allows monitoring the trend of phenomena of great interest, including HAIs. Unfortunately, the decision to use KPIs based on the outcome requires a critical filter on the emerged data, including the degree of awareness of citizens towards specific health-related phenomena that also influence the reporting rate [65]. If the decision to focus the analysis on the rate of claims may lead to limitations, it is also possible to state that this approach provides the basis for an economic assessment of the burden represented by the single items of damage. This could allow clinical risk management decisions to be oriented on the basis of the economic expenditure linked to individual categories; in the future, however, the application of the same methodological criteria for the construction of process KPIs could represent a valuable tool with which to close the loop relating to the improvement of the quality of care.

In any case, the main criticality linked to the application of such a tool to other healthcare facilities is the possible lack of a precise register that contains information on all claims based on time, operating unit, category of claim, the characteristics of the patient, and news of an economic nature linked to the judicial progress of the claim. From this point of view, a valuable tool has been provided in Italy by the provisions of Law no. 24/2017, according to which all healthcare facilities are required to prepare and publish online all litigation data per year [72]; in this way, the easy availability of sensitive data relating to the legal expenses incurred by the structures can be an ally for the processing of such data by specially trained external bodies.

Therefore, our results provide a picture of the “quality of care” delivered by Umberto I General Hospital, Campus Bio-Medico University Hospital Foundation and Agostino Gemelli IRCCS University Hospital Foundation, from 2013 to 2020. Obtained data could be used to compare our hospital performance over time and with different hospitals. In fact, while the formulation of process indicators moves from guidelines analysis and standards identification, outcome indicators are not expected to fulfill any previously defined requirement, simply providing an analytic tool for performance evaluation [72].

Moreover, a limitation of the present analysis is constituted by the lack of consideration of fundamental aspects related to the hospital management of claims, as the economic and/or humanistic point of view; further analyses will be able to demonstrate the definitive suitability of the indicators proposed in this paper for practical use in the hospital setting.

## 5. Conclusions

In this study, the outcome indicators were conceived starting from a retrospective analysis of the medico-legal claims data. Therefore, the study limited itself to portraying the adverse events that resulted in a request for compensation and/or the initiation of legal proceedings. However, we are convinced that this is only a first step. The science of safety in care must aim for professional excellence. It is necessary to go further and identify standardized indices that can reveal adverse events, even if there have been no claims for compensation or the initiation of legal proceedings or a spontaneous report by the health professionals.

As previously stated, Clinical Risk Management represents one of the structural programmatic activities of the healthcare system. The need to identify more suitable tools for a “real” evaluation of different healthcare settings, as well as any possibility for improvement, has led to a series of research strategies that, comparing different organizations, would provide necessary elements to define policies of quality improvement. In this context, the use of indicators in both public and private healthcare realities is destined to become an essential asset for every hospital. Precisely, the practice of health facilities accreditation, as provided for by the National Health Plan in the last decade, responds to the need to select good providers of health services based on qualitative criteria.

This will allow health organizations to effectively monitor verified and unreported adverse events, and allow the possibility of implementing measures and interventions aimed at the safety of medical care and procedures to protect the patient.

## Figures and Tables

**Table 1:** Briefly reported numbers of the selected structures.

Structure	Umberto I General Hospital	Agostino Gemelli University Hospital Foundation	Campus Bio-Medico University Hospital Foundation
Ordinary beds (no.)	1235	1536	296
No. of hospitalizations in the last year	41,000	94,509	28,700 hospitalizations
No. of accesses to the emergency room	Over 140,000	83,419	11,600

**Table 2:** Ideal characteristics of an indicator, according to specific categories.

Dimension	Index	Outcome indicator	Description
Clinical setting	Quality of care	Accidental falls rate	Number of accidental falls/total of claims
		Healthcare-related infections rate	Number of healthcare-related infections/total of claims
		Surgical site infections rate	Number of surgical site infections/total of claims
		Transfusion-related adverse events rate	Number of transfusions-related adverse events/total of claims
		Drug administration-related adverse events rate	Number of drug administration-related adverse events/total of claims
		Birth injuries rate	Number of maternal birth injuries/total of claims
		Maternal deceases after birth rate	Number of maternal deceases after birth/total of claims
		Wrong-site surgery/wrong procedure rate	Number of wrong procedures and wrong-site surgeries/total of claims
		Retained surgical instruments’ rate	Number of cases of retained surgical instruments/total of claims
		Clinical devices malfunction rate	Number of cases related to clinical devices malfunction/total of claims
		Triage error rate	Number of cases related to triage errors/total of claims
		Violence against healthcare workers rate	Number of cases related to violence against healthcare workers/total of claims
		Suicide in medical setting rate	Number of suicides in medical setting/total of claims

## Data Availability

Not available.
